# Ultra-subwavelength thickness for dual/triple-band metamaterial absorber at very low frequency

**DOI:** 10.1038/s41598-018-29896-4

**Published:** 2018-08-02

**Authors:** Bui Xuan Khuyen, Bui Son Tung, Young Ju Kim, Ji Sub Hwang, Ki Won Kim, Joo Yull Rhee, Vu Dinh Lam, Yong Hwan Kim, YoungPak Lee

**Affiliations:** 1 Department of Physics and RINS, Hanyang University, Seoul, Korea; 2 Department of Display Information, Sunmoon University, Asan, Korea; 3 Department of Physics, Sungkyunkwan University, Suwon, Korea; 4 Institute of Materials Science, Vietnam Academy of Science and Technology, Hanoi, Vietnam; 5 Infovion Co., Seoul, Korea

## Abstract

An integrated model utilizing external parasitic capacitors for a dual-band metamaterial perfect absorber (DMPA) is proposed and demonstrated in the UHF radio band. By adjusting the lumped capacitors on a simple meta-surface, the thickness of absorber is reduced to be only 1/378 and 1/320 with respect to the operating wavelength at 305 and 360.5 MHz, respectively. The simulations and the experiments confirm that the DMPA can maintain an absorption over 91% in a wide range of incident angle (up to 55°) and independent of the polarization of incident radiation. Additionally, we examine the integrated model for smaller dual-band absorber and absorption performance at higher frequencies (LTE band). Finally, we consolidate our approach by fabricating an ultrathin triple-band perfect absorber miniaturized to be only 1/591 of the longest operating wavelength. Our work is expected to contribute to the actualization of metamaterial-based devices working at radio frequency.

## Introduction

In recent years, technology has been developed rapidly owing to the advances in material science. Thus, the discovery of new materials with useful properties is very important in developing better technologies. In looking for new exciting materials and effects, an artificial material named “metamaterial” (MM) was realized and has changed our understanding of light-matter interactions in nature^1,2^. The mechanism of these MMs, whose unit cells (so-called meta-atoms) are much smaller than the operating wavelength, is combined with the theory of effective medium^3^. By being able to independently control the effective permittivity [${\varepsilon }_{eff}(\omega )]$ and permeability [${\mu }_{eff}(\omega )$], MMs reveal great potential for the practical applications such as negative refractive index^4–6^, invisibility cloak^7,8^, and perfect lens^9,10^. In 2008, the unique phenomenon of nearly perfect absorption was first observed and realized by Landy *et al*.^11^. Their MM perfect absorber (MPA) had a unit-cell size of λ/30, much smaller than the previous generation of absorbers^12^. Henceforth, MPAs have been studied intensively for frequencies in the radio to the optical range^13–18^. Because of their compatibility with peripheral devices, the MPAs are great candidates for future devices such as bolometers^19^, thermal imagers^20,21^, solar cells^22^, bio-sensors^23^, and high-subwavelength-resolution cameras^24^.

In companion with the rapid growth of telecommunication technology, MPAs have been introduced to realize readability within radio-frequency identification (RFID) systems^25,26^, power imaging^27^, chipless RFID tags^28^ and sub-GHz wireless systems^29^. However, multi-band absorption based on ultrathin MPAs are still an important target to improve the future meta-instruments for telecommunication systems. For this purpose, we introduce and investigate, first of all, an ultrathin dual-band MPA (DMPA) that operates in sync with peripheral capacitors. In addition to functioning in the ultrahigh-frequency (UHF) band (300–3000 MHz), the suggested DMPA has shown the ability to perfectly consume the incoming energy of an electromagnetic (EM) wave with any polarization and wide oblique incidence angles. Furthermore, the proposed integrated model is also effective for scaling-down and flexible structures. In particular, by adding-on lumped capacitors to the basic DMPA, an ultrathin triple-band MPA is also realized and investigated in the radio band.

## Design, Results and Discussion

**Figure 1:**
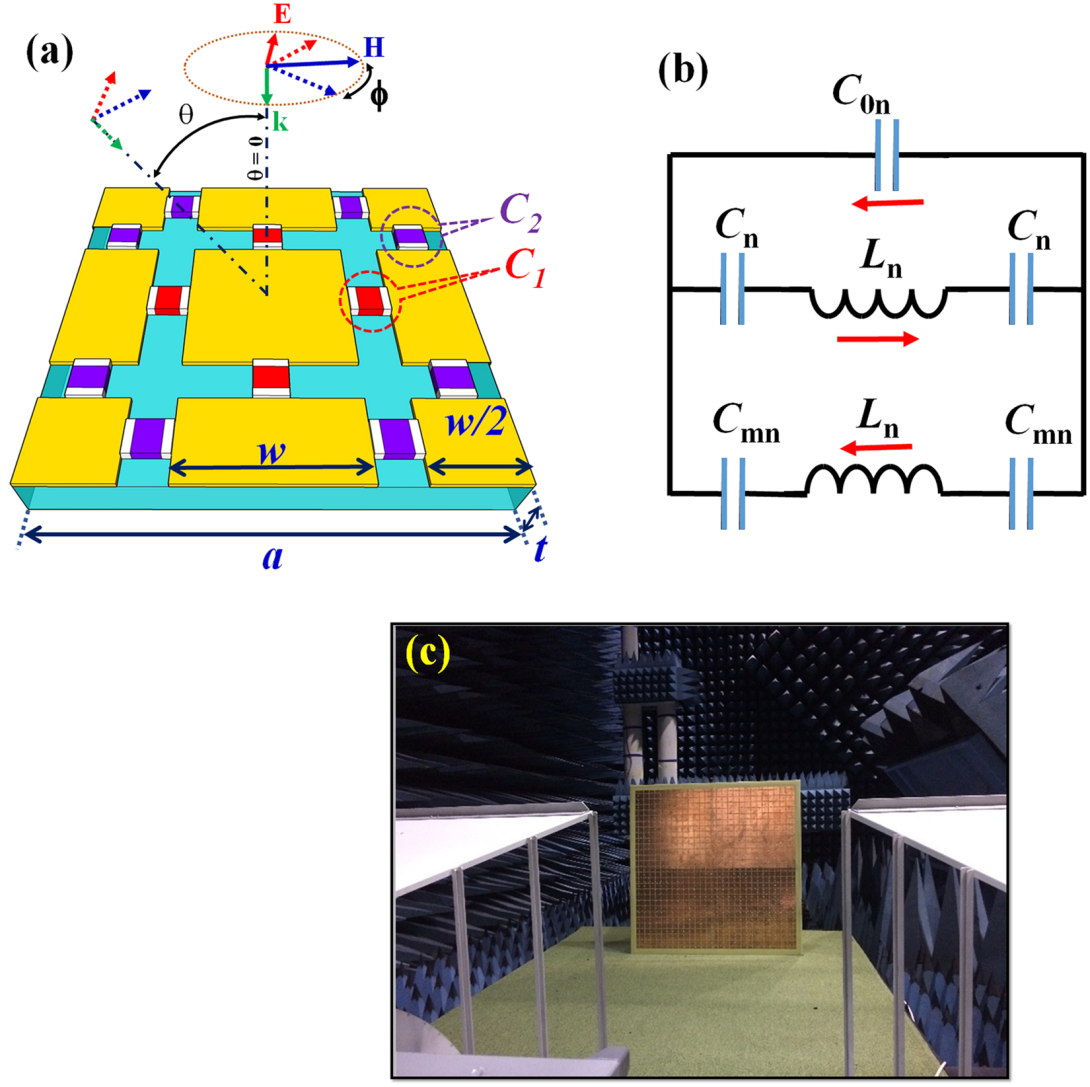
Schematic of the simulation and the measurement for ultrathin DMPA. (**a**) 3-dimensional periodic structure of the unit cell and (**b**) its equivalent LC-circuit model. Red arrows represent the flowing direction of induced currents. (**c**) Experimental configuration of the proposed DMPA with the polarization of EM wave.

The 3-dimensional design (single unit cell with the geometric parameters) for our ultrathin DMPA is presented in Fig. 1(a). The air gaps on the top meta-surface are embedded by the peripheral capacitors at the midpoint of each edge. The top patterned and the bottom continuous copper layers (electric conductivity of σ = 5.8×10^7^S/m, and thickness *t*_*m*_ = 0.036 mm) are separated by a dielectric substrate (thickness of *t*). From the well-known equivalent inductor-capacitor (LC) circuit model for MPA^30,31^, the key idea of our design is to enhance the effective capacitance by adding external lumped capacitors. Consequently, the resonance and the perfect impedance matching are obtained at very low frequencies. Figure 1(b) represents the typical LC-circuit model for the performance of our DMPA. Where *L*_n_ and *C*_mn_ are the intrinsic effective inductance of front and back metallic layers, and the intrinsic effective capacitance between copper-patterned layer and bottom continuous copper plane for the center-patterned series (n = 1) and the other-patterned series (n = 2) along **E** direction, respectively. For each case, the external-lumped capacitor and the mutual effect caused by other capacitors on the meta-surface are denoted as “*C*_n_” and “*C*_0n_” (n = 1, 2), respectively.

It is well-known that the perfect-absorption behavior can be explained by the impedance-matching theory^32^. At each absorption frequency, the effective impedance of MPA is perfectly matched with the free space $(Z=\sqrt{\mu (\omega )/\varepsilon (\omega )}=1)$, which is caused by the electric permittivity *ε*(*ω*) and the magnetic permeability *μ*(*ω*) being equal. The effective impedance can also be expressed in terms of the reflection and the transmission parameters:

1 $$Z=\sqrt{\frac{{(1+{S}_{11}(\omega ))}^{2}-{S}_{21}^{2}(\omega )}{{(1-{S}_{11}(\omega ))}^{2}-{S}_{21}^{2}(\omega )}}.$$

**Figure 2:**
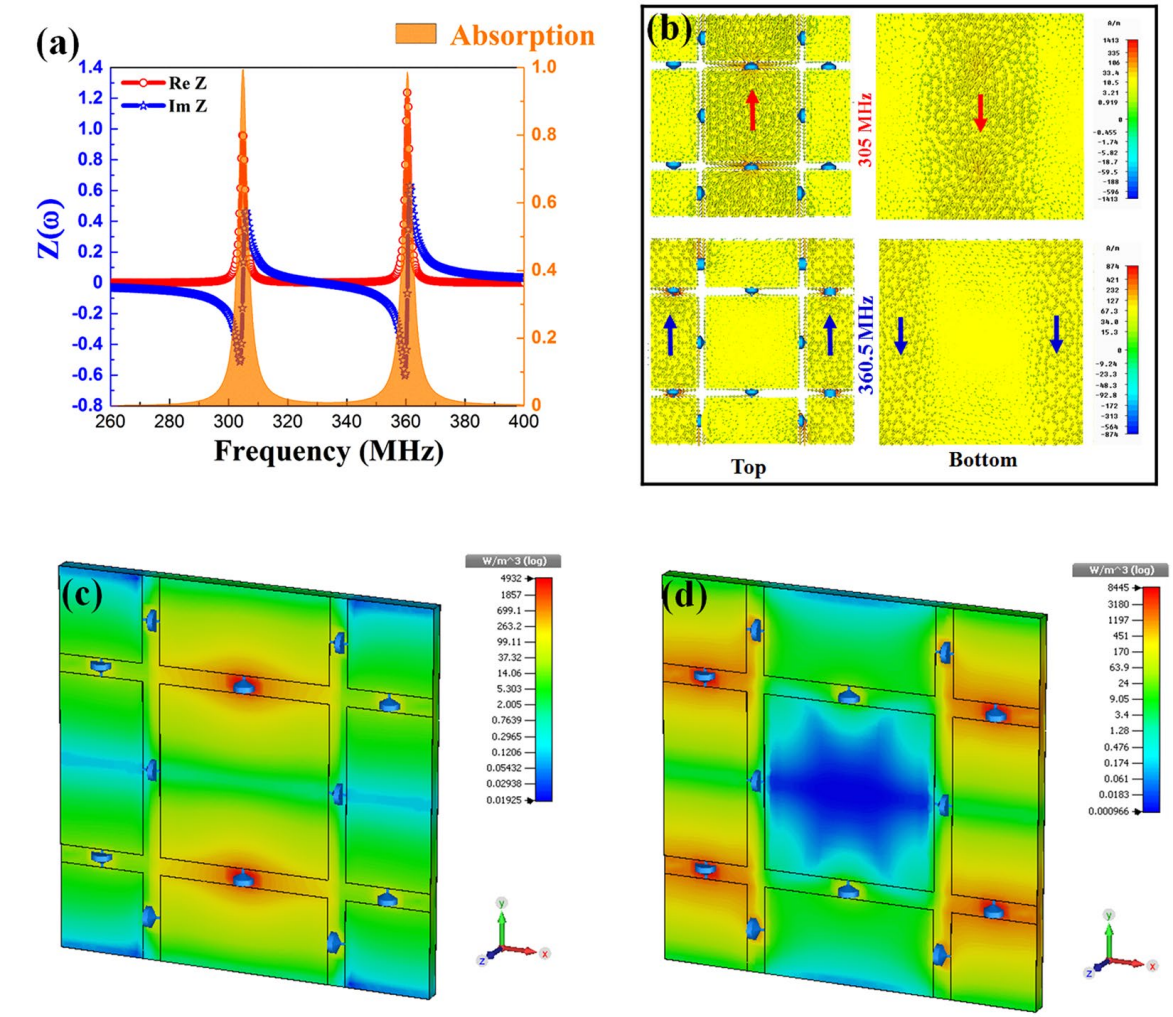
Physical mechanism of dual-band perfect absorption. (**a**) Simulated effective impedance and absorption spectrum of the DMPA. Distributions of (**b**) the induced surface currents on front and back layers. 3-dimensional distributions for the power loss at (**c**) 305 and (d) 360.5 MHz.

For our basic DMPA, FR-4 was chosen as the dielectric substrate (thickness *t* = *2.6* *mm*) with a dielectric constant of 4.3 and a loss tangent of 0.025, and the geometrical parameters of proposed DMPA are optimized as *a* = 64.0 and *w* = 29.0 mm. Capacitors *C*_1_ and *C*_2_ are assigned to have the values of 47.0 and 24.0 pF, respectively. In Fig. 2(a), both extracted real and imaginary parts of the relative impedance are plotted. It is clearly observed that the effective impedance has an imaginary part of zero and a real part of approximately 1.0 at both resonant frequencies (305 and 360.5 MHz). In other words, there is no reflected wave for these frequencies, due to the fact that the impedance of DMPA matches well with that of the ambient environment. Simultaneously, this phenomenon is associated with the intrinsic resonances to effectively consume the energy of incoming EM wave inside the DMPA. As revealed in Fig. 2(a), a nearly-perfect dual-absorption peak at 305 and 360.5 MHz (absorption of 99.5% and 98.8%, respectively) is achieved.

The mechanism of energy dissipation can be visualized as the simulated surface-current, magnetic-energy and power-loss-density distributions at two absorption frequencies [as presented in Fig. 2(b–d)]. Figure 2(b) shows the induced currents flowing on the meta-surfaces at 305 and 360.5 MHz. The induced surface currents, which are caused by excitation along the electric field, accumulate in a bottom-up direction (indicated by red arrows) on the center patterns and the opposite top-down direction in the continuous copper layer, for the first absorption peak. In case of the second absorption peak, another kind of induced currents (their directions are indicated by blue arrows) are mainly concentrated on the left and the right parts of unit cell. Consequently, these anti-parallel currents, which indicate two magnetic resonances, are formed separately within the DMPA. In other words, the perfect absorption originates from these magnetic resonances where the perfect impedance matching arises at 305 and 360.5 MHz.

Figure 2(c,d) show that the induced power loss (dielectric loss) of the EM wave is concentrated in areas, consistent with the aforementioned induced currents. Evidently, the energy of incoming EM wave is completely consumed inside the absorber. Along the external electric-field direction, the power loss is mainly located around the center series of capacitors *C*_1_ (at 305 MHz), and around the left and right series of capacitors *C*_2_ (at 360.5 MHz). In other words, a center-patterned series (containing capacitors *C*_1_) plays a key role in producing the lower perfect absorption peak. Separately, the other-patterned series (containing capacitors *C*_2_) produces primarily the higher one. This analysis is the basis to build a comprehensive equivalent LC-circuit model in Fig. 1(b). Besides, the coupling caused by surrounding capacitors (*C*_0n_) to the main absorption frequency is also expressed clearly as Fig. S1(b,d) in Supplementary, where the absorption frequencies for dual-band MPA are derived by Eq. (S2) and shown in Table S1. It can be seen that the calculated and the simulated absorption frequencies are in good agreement, since the low and the high absorption frequencies are calculated as 304.4 and 361.9 MHz, respectively. From this LC-circuit model, our design can be applied for tunable applications in the UHF band if a variable capacitor is integrated instead of the normal one.

**Figure 3:**
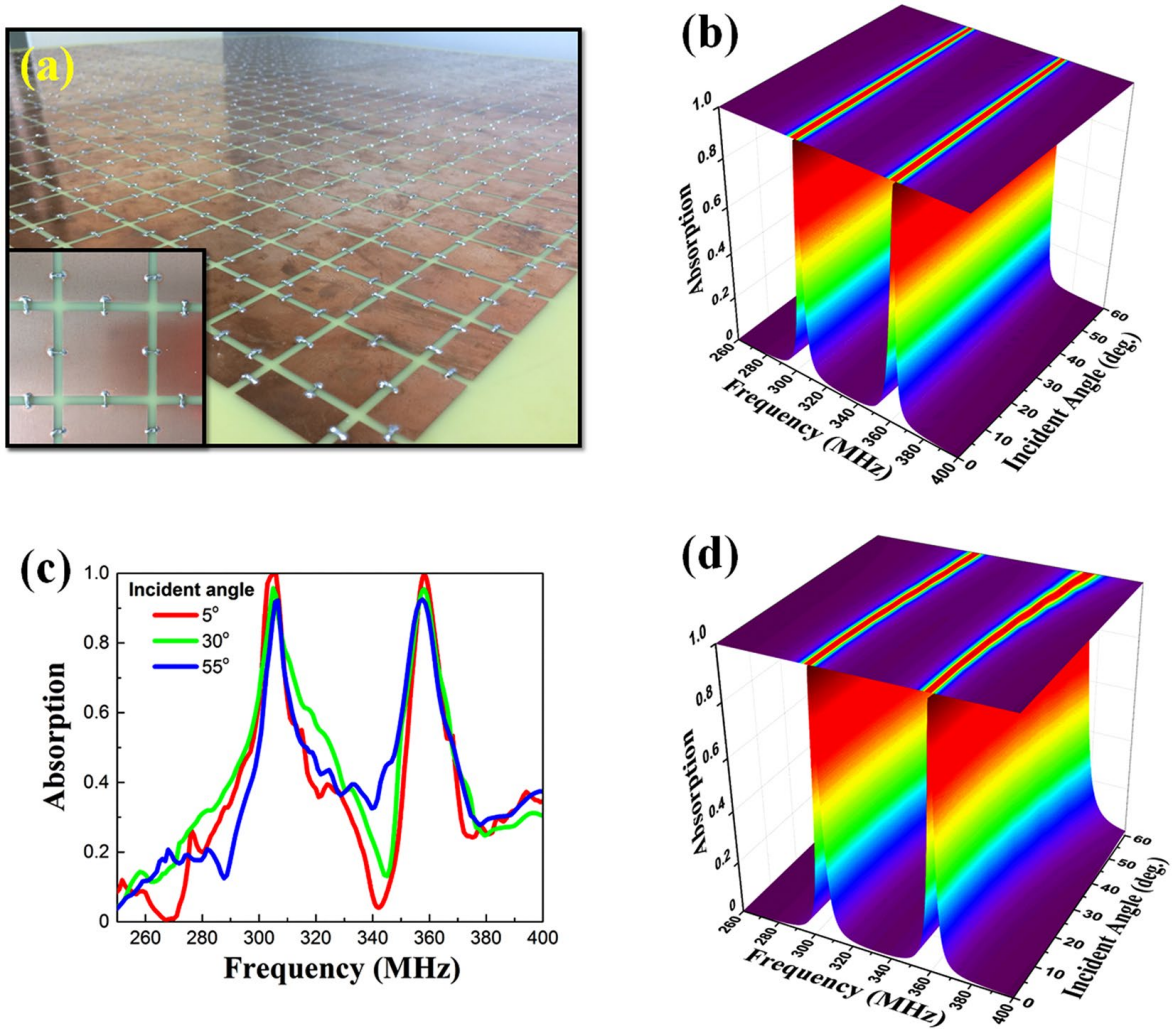
Performance of the basic DMPA in wide range of incident angle. (**a**) Fabricated DMPA (thickness *t* = 2.6 *mm*). (**b**) Simulated and (**c**) measured absorption spectra according to the incident angle of EM wave for the TE polarization. (**d**) Evolution of the simulated absorption spectra for the TM polarization.

The dependence of absorption on the oblique angle of incidence (*θ*) was investigated to evaluate the operational capability. Remarkably, as presented in Fig. 3(b), the absorption of 99.5% (at 305 MHz) and 98.8% (at 360.5 MHz) when *θ* = 0 falls only slightly to 91.1% and 97.1% when *θ* = 55° for the transverse-electric (TE) polarization. In this case, the peak-absorption frequencies are nearly unchanged by *θ.* For the fabricated sample [Fig. 3(a)], the evolution of measured absorption data [Fig. 3(c)] is in good agreement with the simulated spectra. When *θ* = 5°, the absorption peaks are located at 304.9 and 358.5 MHz with an absorption of 99.7% and 99.5%, respectively. For an incident angle of 55°, the measured absorption is maintained to be 91.7% and 92.5% for the lower and the higher frequency, respectively. In comparison with the currently-published DMPAs^33–35^, the smaller ratios of DMPA thickness *t* with respect to the lower and the higher operational wavelength λ are accomplished: *t* = only *λ*/378 and *λ*/321, respectively. A short periodicity with respect to the wavelength is also obtained to be *a* = *λ*/15 at 305 MHz, for example. In case of the transverse-magnetic (TM) polarization at *θ* = 55°, as in Fig. 3(d), the dual-band absorption remains over 90% and both peaks exhibit a slight blue-shift to 313.8 and 371.5 MHz. This behavior is consistent with the observation in earlier works^36,37^. In other words, the designed DMPA conserves well the high absorption in a wide range of incident angles.

**Figure 4:**
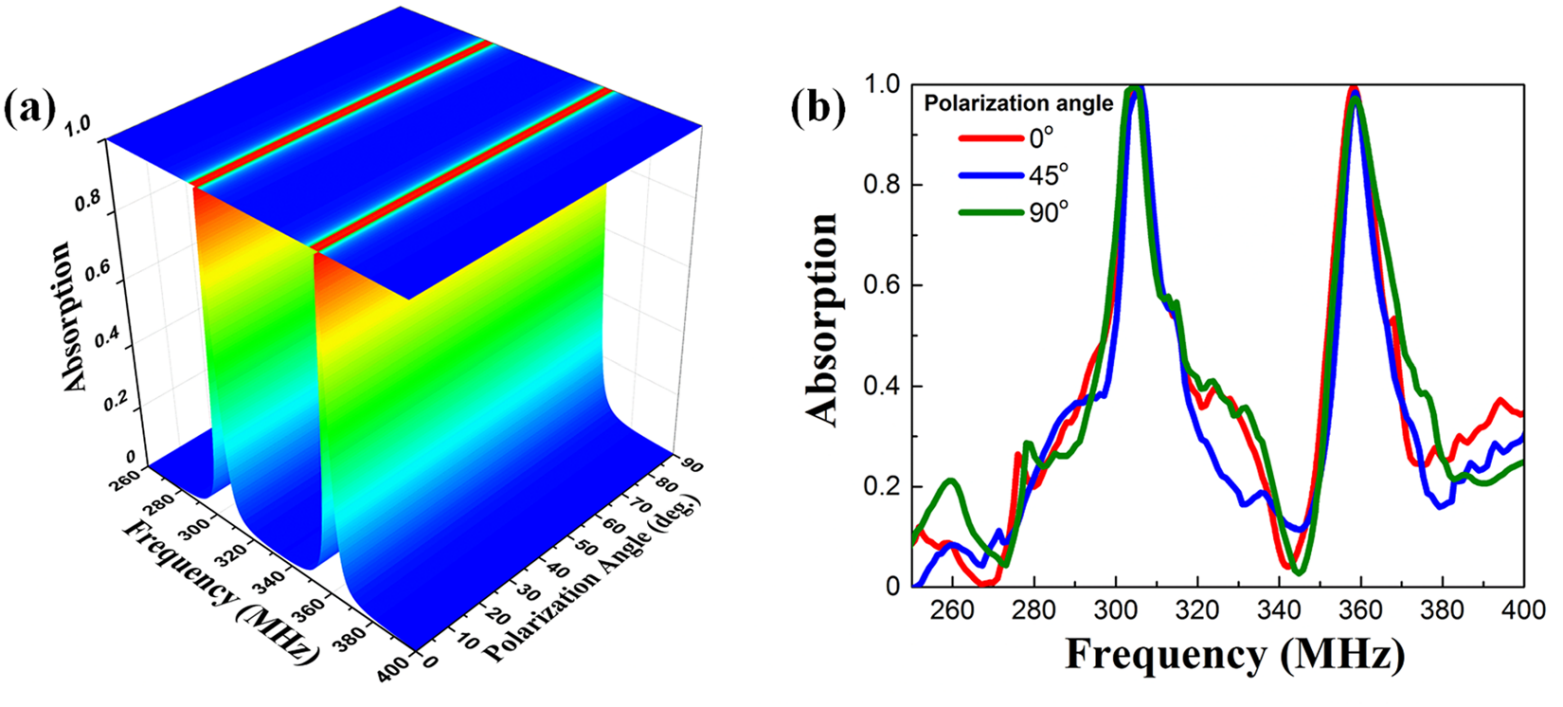
Polarization-independent behavior of the basic DMPA. (**a**) Simulated and (**b**) measured absorption spectra according to polarization angle ϕ.

Furthermore, by exploiting the symmetric structure, as shown in Fig. 4(a), the ultrathin DMPA exhibits the polarization-insensitive behavior. The dual absorption (simulated absorption over 99.5% and 98.8%, respectively, at the lower and the higher frequency) is conserved for the overall polarization angles from 0 to 90° at the normal-incident wave. Correspondingly, the measured absorptions are above 99.6% (at 304.9 MHz) and 97.1% (at 358.5 MHz) for all the polarization angles from 0 to 90° [Fig. 4(b)]. It can be noted that, similar to the previous measurements^38^, the experimental absorption bandwidths are slightly wider than the simulated ones.

**Figure 5:**
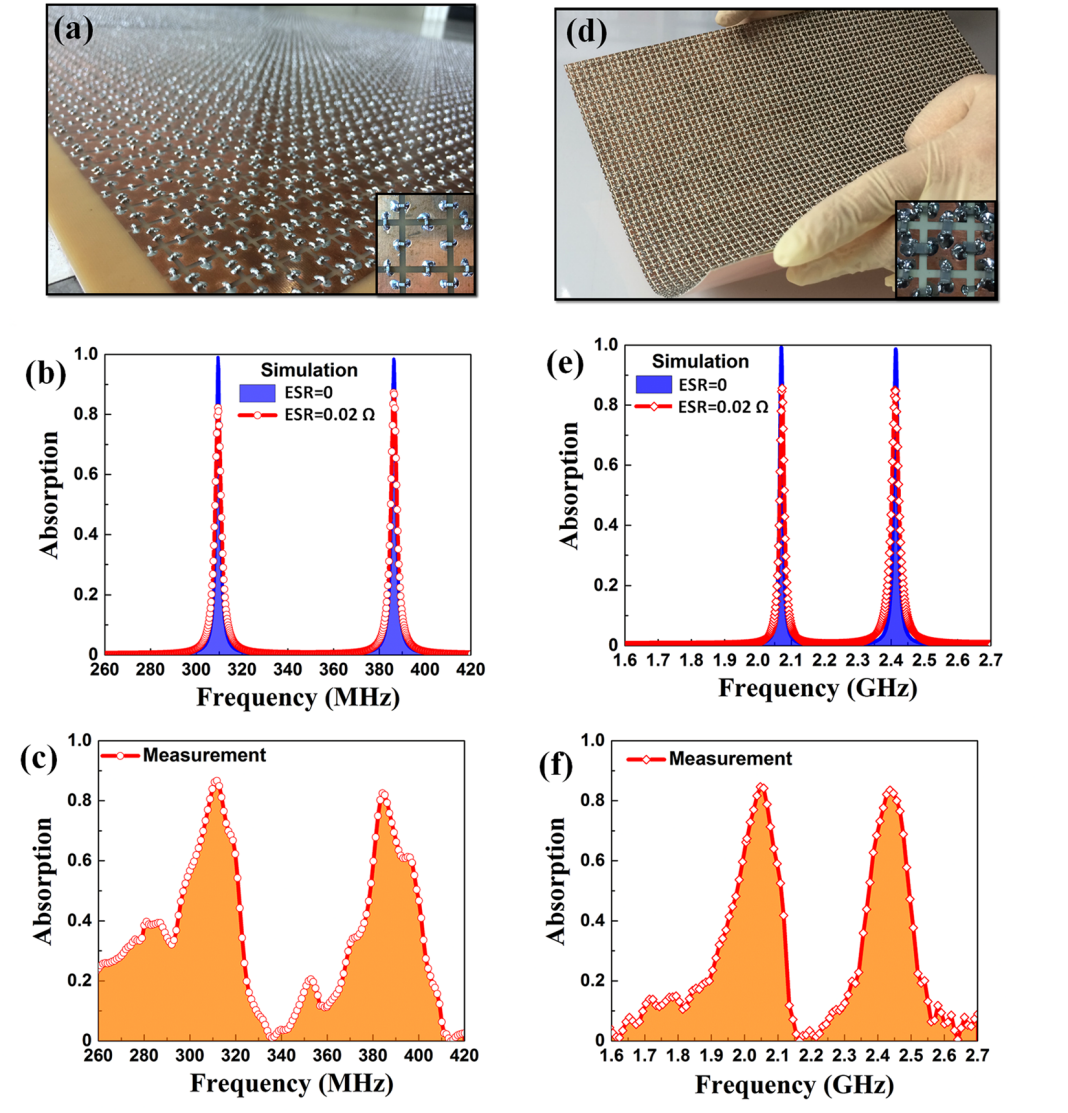
Application of the basic-DMPA model for the smaller size and the higher operational frequency. (**a**) Fabricated sample, (**b**) simulated and (**c**) measured absorption spectra. (**d**) Scaled-down structure for high-frequency DMPA, (**e**) simulated and (**f**) measured absorption spectra. Bottom insets of (**a**) and (**d**) are the enlarged view of single unit cell.

In addition, we have assessed the applied scope of our model by carrying out two different investigations. The first one is to reduce the size of absorber while keeping the same operational frequency. The second one is to scale down the size of absorber to make a higher operational frequency in the long-term-evolution (LTE) band. In the first case, the geometrical parameters of initial DMPA are reduced to be *w* = 10.0, *a* = 23.0, and *t* = 1.2 mm. The initial capacitors *C*_1_ and *C*_2_ are modified as *C*_*1*_ = 100.0 and *C*_2_ = 47.0 pF, respectively. Note that, in order to maintain the same frequency region while reducing the size of absorber, the value of capacitance should be higher. Consequently, the simulated results reveal nearly-100% absorption peaks at 309.5 and 386.2 MHz at the normal incidence [Fig. 5(b)]. The corresponding fabricated DMPA [Fig. 5(a)] exhibits two absorption peaks at 311.8 MHz (absorption of 87%) and 384.2 MHz (absorption of 83%). Here, the absorber is miniaturized to be *λ*/800 and *λ*/42 for thickness and unit-cell width, respectively, with respect to the longer wavelength.

In the second case (LTE region), the geometrical parameters of initial DMPA are nearly linearly scaled down to be *a* = 12.0 and *w* = 5.0 mm. For better applicability, instead of FR-4 substrate, a high-flexible dielectric layer named Ultralam-3850HT (thickness *t* = 0.35 mm) is selected with its dielectric constant and loss tangent being 3.14 and 0.002, respectively. The initial capacitors are suitably changed so that *C*_1_ = 3.6 and *C*_2_ = 2.0 pF, respectively. The simulated result yields an absorption over 98% at 2.07 and 2.4 GHz [Fig. 5(e)]. Two measured absorption peaks are detected at 2.05 GHz (absorption of 84.6%) and 2.43 GHz (absorption of 83.5%) as plotted in Fig. 5(f). The ratios of thickness *t* and lattice constant *a* of flexible DMPA to the wavelength λ at 2.05 GHz are realized as *t* = *λ*/417 and *a* = *λ*/12.

For these two applied cases, although the positions of experimental dual peaks are in good agreement with the simulated ones, the absorption magnitudes are lower than 90% for the experimental measurements. These deviations are anticipated by the impaired resonance and the impedance mismatching due to influence of the real capacitors on the small structure. An actual capacitor can be modeled as an ideal capacitor in series with an equivalent series resistance (ESR). The ESR value for low-loss ceramic capacitors is less than 0.1 Ohms, which do not significantly affect the initial absorption in Fig. 2(a). In contrast, when *C*_1_ and *C*_2_ were built in, they had an ERS of 0.02 Ohms, which causes the absorption [Fig. 5(b,e)] to be reduced below 90% for higher frequencies and smaller DMPA. This observation is matched well with the measured absorption. Normally, the impedance matching [*ε*(*ω*) = *μ*(*ω*)] can occur in only a narrow frequency range by properly superposing the magnetic resonance and the plasma behavior^39,40^. In our structure, the impedance matching might be affected by the highly-dense elements of external capacitors and the soldering material on the meta-surface. For the miniaturized absorbers, the area of capacitors is comparable to that of the pattern. Therefore, the loading of an intrinsic-loss component inside the practical capacitors, which can be ignored for the basic DMPA in Fig. 3(a) because the area of pattern is much wider than that of capacitors, makes the impedance-matching condition unsatisfied for the second and the third DMPAs. To improve the impedance-matching behavior, the integration with smaller lumped components by using better soldering techniques is necessary in fabrication.

**Figure 6:**
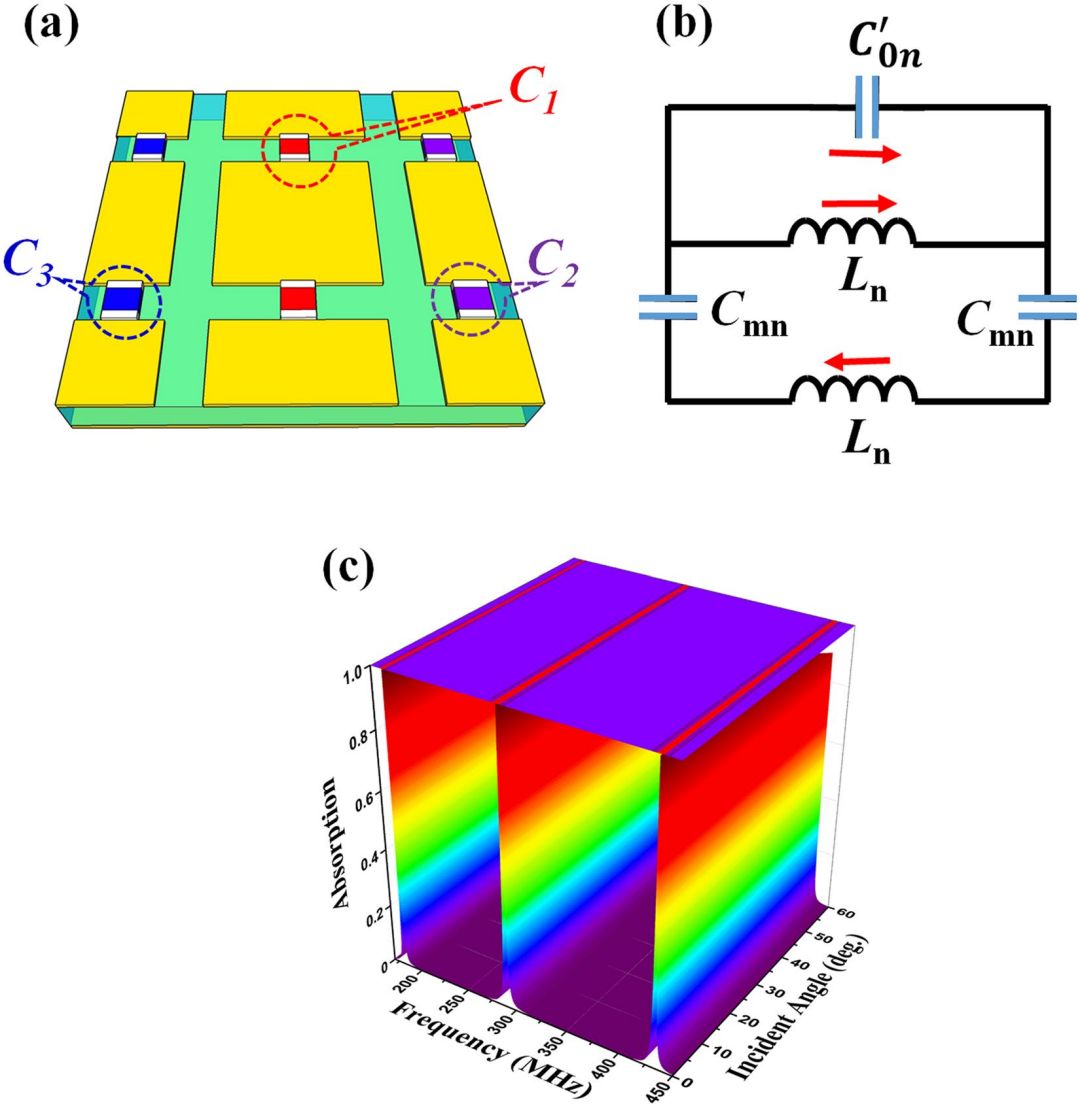
Extension to the triple-band MPA. (**a**) 3-dimensional schematic of the unit cell and (**b**) its equivalent LC-circuit model. (**c**) Simulated absorption spectra according to the incident angle of EM wave for triple-band MPA.

Finally, in order to further consolidate our method, a triple-band MPA is investigated by exploiting an add-on capacitor [Fig. 6(a)]. According to the aforementioned analyses (Fig. 2 and Fig. S1 in Supplementary), the strong magnetic resonance and the corresponding impedance matching are demonstrated well by the separate parts of structure (resonance patterns in sync with peripheral capacitors). Therefore, by rearranging the basic meta-surface in Fig. 6(a), it is expected that the third magnetic resonance should be induced by add-on capacitor (*C*_3_ = 120.0 pF) to sync with original two capacitors (*C*_1_ = 47.0 and *C*_2_ = 24.0 pF). In this case, the thickness of FR-4 is optimized as *t* = 2.8 mm and the other parameters are fixed. To confirm this idea, we can approximately calculate three absorption frequencies through a comprehensive equivalent LC-circuit model in Fig. 6(b). There *L*_n_ and *C*_mn_ are the intrinsic-effective inductance of front and back metallic layers, and the intrinsic-effective capacitance between copper-patterned layer and bottom continuous copper plane, respectively, in the center-square series (n = 1), the right-rectangular series (n = 2) and the left-rectangular series (n = 3) along **E** direction. For each case, the external-lumped capacitance and the mutual effect caused by other capacitors on the meta-surface are unified into only one capacitor “*C*′_0n_” (n = 1, 2, 3), which is expressed clearly as Figs S2(d), S3(d) and S4(d) in Supplementary. Consequently, the absorption frequencies for triple-band MPA are derived by Eq. (S8) and shown in Table S2. The absorption frequencies are expected to be 186.9, 286.0 and 435.5 MHz. In the simulation, a triple-band perfect absorption is also realized in Fig. 6(c). At the normal incidence, three absorption peaks of 99.9%, 99.8% and 99.5% are achieved at 181.2, 290.4 and 432.4 MHz, respectively. It can be seen that the calculated and the simulated absorption frequencies are in good agreement. In particular, when the incident angle is increased to be 55°, the simulated absorption remains over 91.5% for these respective frequencies. In comparison with the other structures in this paper^41–44^, our triple-band MPA has the smallest thickness with respect to the operating wavelengths (*t* = *λ*/591, *λ*/368 and *λ*/247 corresponding to 181.2, 290.4 and 432.4 MHz, respectively).

## Conclusions

New types of ultrathin DMPAs were investigated by experiment, simulation and calculation in the UHF region. By integrating external capacitors to tune the magnetic resonance at very low frequencies, the thickness of dual-band absorber was efficiently miniaturized to be 0.0026λ (at 304.9 MHz) and 0.0031λ (at 358.5 MHz). The practical operation (absorption over 91% in the TE and the TM polarizations) of this DMPA was presented for a wide range of incident angles (up to 55°). In addition, the simulation and the measurement also show that the dual-band absorption is polarization-independent at the normal incidence of EM wave. Further experiments reveal that there are limitations of our model for the even smaller size of absorber or the even higher operational frequency. We expected that these obstacles could be solved by using smaller capacitors and better soldering techniques. We also developed a tri-element-integrated model that recognized an ultrathin triple-band MPA (thickness of 0.0017λ at 181.2 MHz) working in a lower frequency region. Thus, we believe that our ultra-subwavelength structures are useful and can be applied to future meta-devices in the radio band.

## Methods

### Simulations

In our work, the simulations were conducted through a finite-integration package, CST Microwave Studio^45^. In general, the absorption can be calculated as $A(\omega )=1-|{S}_{21}(\omega ){|}^{2}-|{S}_{11}(\omega ){|}^{2}$, where *S*_11_(*ω*) and *S*_21_(*ω*) are the reflection and the transmission parameters, respectively. Since *S*_21_(*ω*) = 0 (since the back layer is a continuous copper layer), the absorption is simplified as $A(\omega )=1-|{S}_{11}(\omega ){|}^{2}$. The cross-polarized reflection was examined as less than −30 dB for our designs at all the incident and polarization angles.

### Fabrication

To adequately include the main beam of transmitting antennas, the DMPAs were 1000 × 1000 mm^2^ for two samples working in the MHz range [Figs 3(a) and 5(a)], and 400 × 400 mm^2^ for the flexible sample working in the GHz range [Fig. 5(d)]. Their meta-surfaces were precisely etched by the standard printed-circuit-board technique (photolithography). A total of 37,956 chip capacitors were soldered by using the soldering process. These chip capacitors are multilayer ceramic capacitors (MLCC-SMD/SMT) that are suitable for operating at radio frequency.

### Measurement

The experimental configuration is arranged in a microwave anechoic chamber by using a free-space measurement method, as arranged in Fig. 1(b). The reflected coefficients were measured by using a Hewlett-Packard E8362B network analyzer (the operational frequencies from 200 MHz to 20 GHz). In order to allow the EM waves to radiate sufficiently and to guarantee the far-field condition of antenna in the microwave anechoic chamber, the distances between antennas to samples are kept at 6.8 and 2.5 m for the measurement in the MHz and the GHz range, respectively. The incident angles of EM waves (from 5° to 55°) were precisely established by the aperture of two horn antennas and the distance from the middle point of these antennas to the sample.

## Electronic supplementary material

Supplementary Information
