# Supplementary material for: Ultra-subwavelength thickness for dual/triple-band metamaterial absorber at very low frequency
Source: Sci Rep. 2018 Aug 2;8:11632. doi: 10.1038/s41598-018-29896-4 (PMC6072771; doi:10.1038/s41598-018-29896-4)
Supplement: Supplementary file 1 — Supplementary Information [file 41598_2018_29896_MOESM1_ESM.docx]

**Supplementary Information for “Ultra-subwavelength thickness for dual/triple-band metamaterial absorber at very low frequency”**

Bui Xuan Khuyen, Bui Son Tung, Young Ju Kim, Ji Sub Hwang, Ki Won Kim, Joo Yull Rhee, Vu Dinh Lam, Yong Hwan Kim, and YoungPak Lee

Here, in this Supplementary Information, we present details on the theoretical calculations for the equivalent inductor-capacitor (LC) circuit model of dual-band absorption [Fig.1(b)] and triple-band absorption [Fig. 6(b)].

**I. Equivalent circuit model for dual-band metamaterial perfect absorber**

As observed in Fig. 2, the underlying physics of proposed dual-band metamaterial perfect absorber (MPA) is the magnetic resonant phenomenon. Therefore, the absorption frequencies can be quantitatively defined by the equivalent inductor-capacitor (LC) circuit model. The resistor element is ignored for simplicity in our calculations. Based on the distributions of surface-current [Fig. 2(b)] and induced magnetic field at low and high absorption frequencies [Figs. S1(a) and S1(c), respectively], the LC circuits can be made correspondingly for the center-patterned series (n = 1) and the other-patterned series (n = 2) as shown in Figs. S1(b) and S1(d). For these LC-circuit models, the mutual coupling of surrounding capacitors on the meta-surface with the main absorption frequencies is also considered. It can be noted that these two circuits can be regarded as a comprehensive one [as shown in Fig. 1(b)], where the effective capacitance “*C*_0n_” (n = 1, 2) represents the mutual effect caused by the different arrangement of surrounding capacitors on the meta-surface. The total impedance of oscillator circuit in Fig. 1(b) is given by

$$Z_{n}=\frac{1}{i\omega_{n}}\left[ \frac{2-\omega_{n}^{2}L_{n}C_{mn}}{2C_{0n}+C_{mn}-\omega_{n}^{2}L_{n}C_{mn}C_{0n}} \right]+\frac{2}{i\omega_{n}C_{n}}+i\omega_{n}L_{n} \left( n=1, 2 \right). (S1)$$

Consequently, the magnetic resonant frequency can be derived by the condition of Im(*Z*) = 0 to be

$$f_{n}=\frac{\omega_{n}}{2\pi}=\frac{1}{2\pi}\sqrt{\frac{1}{2}\left[ \frac{B}{A}-\sqrt{\left( \frac{B}{A} \right)^{2}-4\left( \frac{C}{A} \right)} \right]}, (S2)$$

where

$$A=L_{n}^{2}{C_{n}C}_{mn}C_{0n},$$

$B=L_{n}\left[ \left( 2C_{0n}+C_{mn} \right)C_{n}+2C_{mn}C_{0n}+C_{n}C_{mn} \right],$ (S3)

$$C=2\left[ 2C_{0n}+C_{mn}+C_{n} \right].$$

Generally, the effective inductance (*L_n_*) of front and back metallic layers is approximately expressed by^1^

$$L_{n}=\frac{\mu_{0}l_{n}}{2\pi}\left[ \ln\frac{2l_{n}}{w_{n}}+0.5+\frac{w_{n}}{{3l}_{n}}-\frac{w_{n}^{2}}{24l_{n}^{2}} \right]. (S4)$$

The intrinsic interaction between copper-patterned layer and bottom continuous copper plane for the center series (*n* = 1) or the other series (*n* = 2) is represented by the contribution of effective capacitance (*C_mn_*), which is given by

$$C_{mn}=\frac{\varepsilon\varepsilon_{0}\alpha_{n}}{t}\left[ w_{n}l_{n} \right]. \left( S5 \right)$$

For the Eqs. above, *t* is thickness of dielectric layer, *l*_n_ and *w*­_n_ are effective length and width of metallic plate on the meta-surface (along **E** direction), respectively. *μ*_0_ and *ε*_0_ are the free-space permeability and permittivity, respectively. *ε* is the permittivity of FR-4 and *α*_n_ is a geometrical factor, 0.4 ≤ *α*_n_ ≤ 0.5. It can be noted that the value of α_n_ in our structure slightly differs from that of the isotropic metamaterial structures^2,3^.

From the arrangements in Figs. S1(b) and S1(d), the corresponding effective capacitance *C*_01_ and *C*_02_ can be calculated by

| $C_{0n}=C_{01}\approx\frac{C_{2}}{2} (n=1)$, | (S6) |
| --- | --- |
| ${C_{0n}=C}_{02}\approx\left( \frac{2}{C_{1}}+\frac{2}{C_{2}} \right)^{-1} (n=2)$. |  |


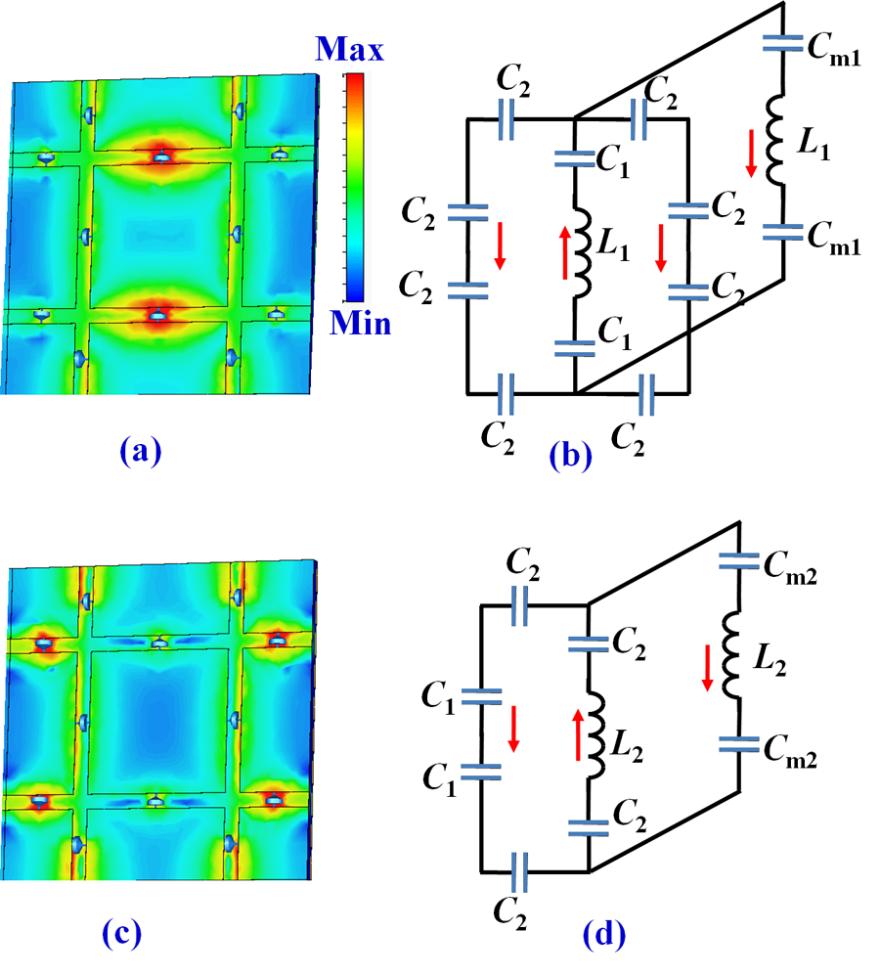


**Figure S1.** Simulated distribution of induced magnetic field inside the DMPA structure at (a) low and (c) high absorption frequencies. The corresponding LC-circuit oscillator models for (b) low and (d) high absorption frequencies.

By applying Eqs. (S1)-(S6), the derived results for the equivalent circuit models in Figs. S1(b) and S1(d) are shown in Table S1.

**Table S1.** **Calculated absorption frequencies for dual-band MPA.**

| Number of modes | *w_n_*  (mm) | *l_n_*  (mm) | *C*_n_  (pF) | *C*_0n_ (pF) | *C*_mn_  (pF) | *L*_n_  (nH) | *α*_n_ | *f*_n_  (MHz) |
| --- | --- | --- | --- | --- | --- | --- | --- | --- |
| n = 1 | 29 | 58 | 47 | 12 | 11.08 | 23.69 | 0.45 | 304.4 |
| n = 2 | 14.5 | 58 | 24 | 7.94 | 5.66 | 30.85 | 0.46 | 361.9 |

**II. Extended LC-circuit model for tripple-band MPA.**

Similarly, three absorption frequencies can be quantitatively defined by the equivalent LC-circuit model in Fig. 6(b), whose total impedance is given by

$$Z_{n}^{'}=\frac{i\omega_{n}^{'}L_{n}}{1-\omega_{n}^{'2}L_{n}C_{0n}^{'}}+\frac{2}{i\omega_{n}^{'}C_{mn}}+i\omega_{n}^{'}L_{n} \left( n=1, 2, 3 \right). (S7)$$

Consequently, the magnetic resonant frequency can be simply expressed as

$$f_{n}^{'}=\frac{\omega_{n}^{'}}{2\pi}=\frac{1}{2\pi}\sqrt{\frac{C_{mn}+C_{0n}^{'}-\left( {C_{mn}^{2}+C'}_{0n}^{2} \right)^{1/2}}{L_{n}C_{mn}C_{0n}^{'}}}. (S8)$$

For Eqs. (S7) and (S8), *L*_n_ and *C*_mn_ are intrinsic-effective inductance and capacitance corresponding to a center-patterned square (if n = 1), a right-patterned rectangular (if n = 2) and a left-patterned rectangular (if n = 3) along **E** direction. It can be noted that capacitance “$C_{0n}^{'}$” (n = 1, 2, 3) can be calculated separately in different LC-circuits made via distributions of the surface current and the induced magnetic field inside the triple-band MPA, as indicated in Figs. S2-S4.

For the middle absorption peak, the magnetic resonance is principally caused by the anti-parallel surface currents between center-patterned series integrated by *C*_1_ and bottom metallic layers. However, these induced-surface currents not only flow significantly in the left-patterned series integrated by *C*_1_, but also partly through the series of adjacent capacitors *C*_3_ and *C*_2_ [Figs. S2(a)-S2(c)]. Consequently, from Fig. S2(d), the value of capacitor “$C_{0n}^{'}$” is calculated by Eq. (S9) below.

$$C_{0n}^{'}=C_{01}^{'}\approx\left( \frac{2}{C_{2}}+\frac{2}{C_{3}} \right)^{-1}+\frac{C_{1}}{2} \left( n=1 \right). (S9)$$

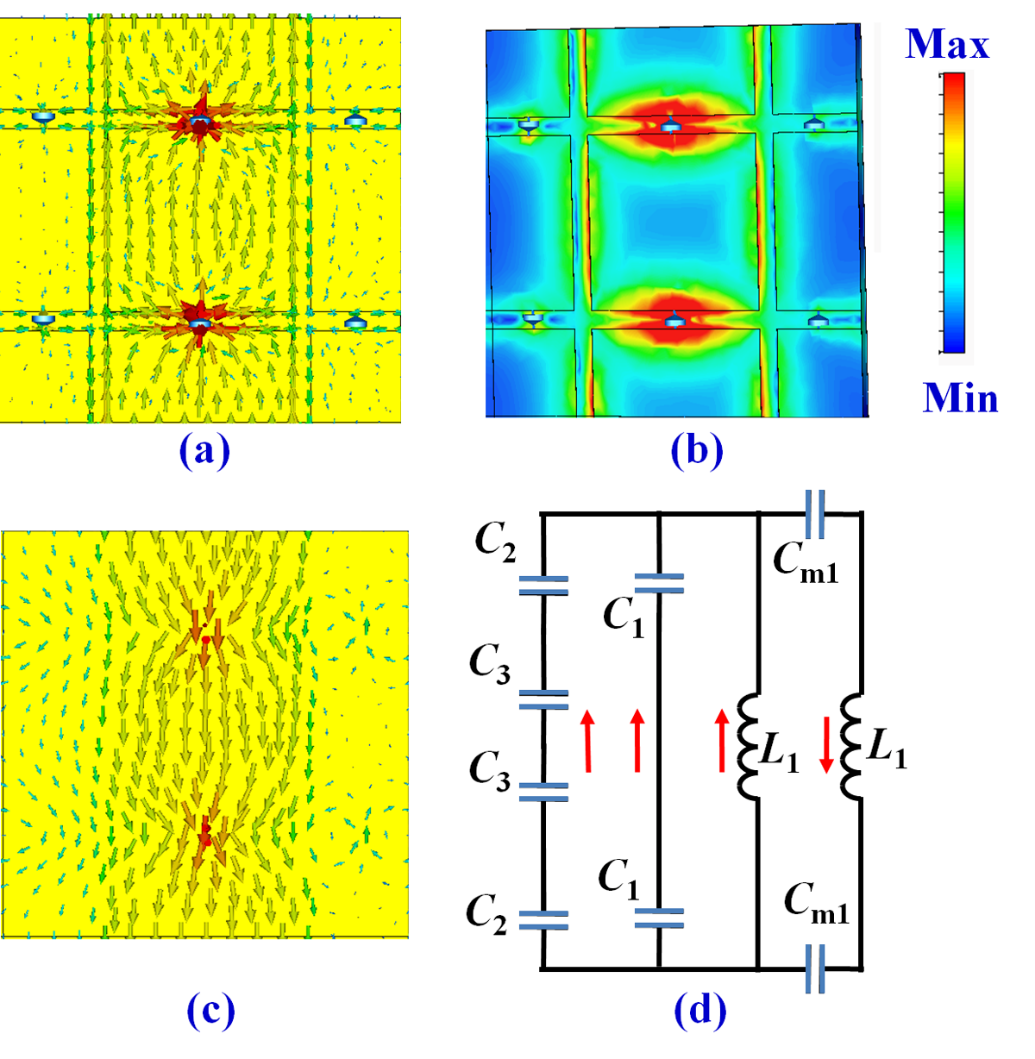


**Figure S2.** Distribution of induced surface currents on (a) front and (c) back metallic layers, and (b) induced magnetic field at middle absorption frequency. (d) Reduced equivalent circuit model for middle resonance (n = 1).

In case of the highest absorption peak, the magnetic resonance is mainly caused by the anti-parallel surface currents between right-patterned series integrated by *C*_2_ and bottom metallic layers. Since these induced surface currents flow significantly in the left-patterned series integrated by *C*_2_ and partly through the series of adjacent capacitor *C*_3_ [Figs. S3(a)-(c)]. From Fig. S3(d), consequently, the value capacitor “$C_{0n}^{'}$” is presented by

$$C_{0n}^{'}=C_{02}^{'}\approx\left( \frac{2}{C_{2}}+\frac{2}{C_{3}} \right)^{-1} \left( n=2 \right). (S10)$$

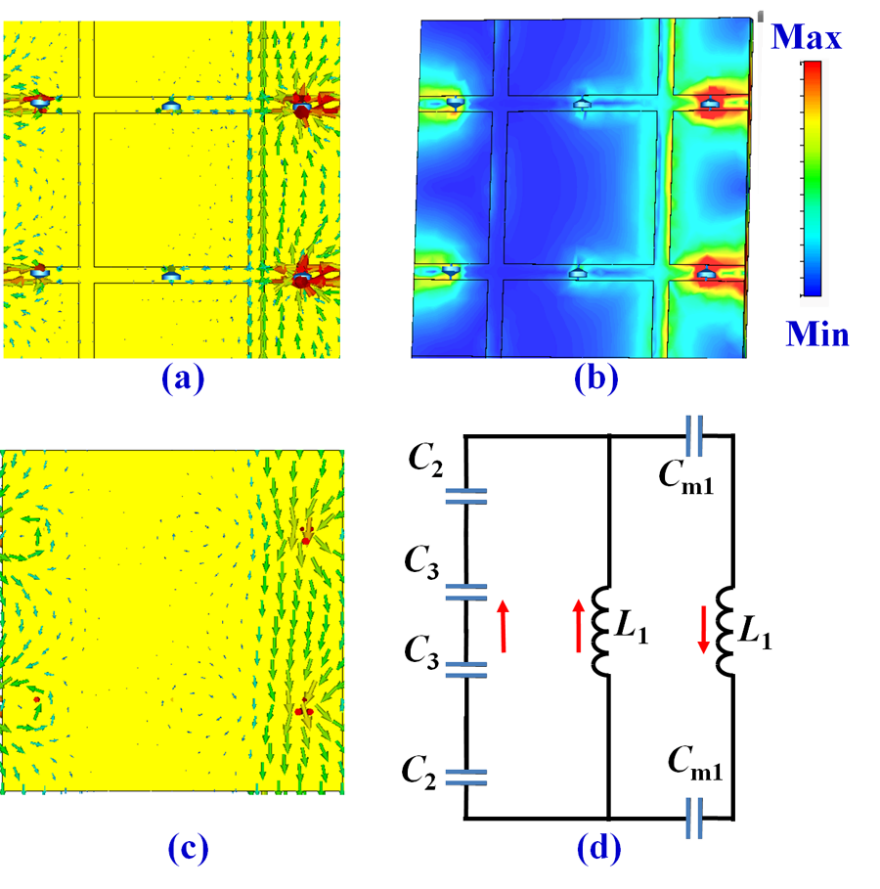


**Figure S3.** Distribution of induced surface currents on (a) front and (c) back metallic layers, and (b) induced magnetic field at the highest absorption frequency. (d) Reduced equivalent circuit model for the highest resonance (n = 2).

In case of the lowest absorption peak, obviously, the magnetic resonance is caused by the anti-parallel surface currents on the left-patterned series and the bottom metallic layers [Figs. S4(a)-(c)]. Since these induced currents flow only though the left-patterned series integrated by capacitor *C*_3_. Therefore, the effect caused by other capacitors (*C*_2_ or *C*_1_) to the lowest absorption frequency can be neglected.

$$C_{0n}^{'}=C_{03}^{'}\approx\frac{C_{3}}{2} \left( n=3 \right). (S11)$$

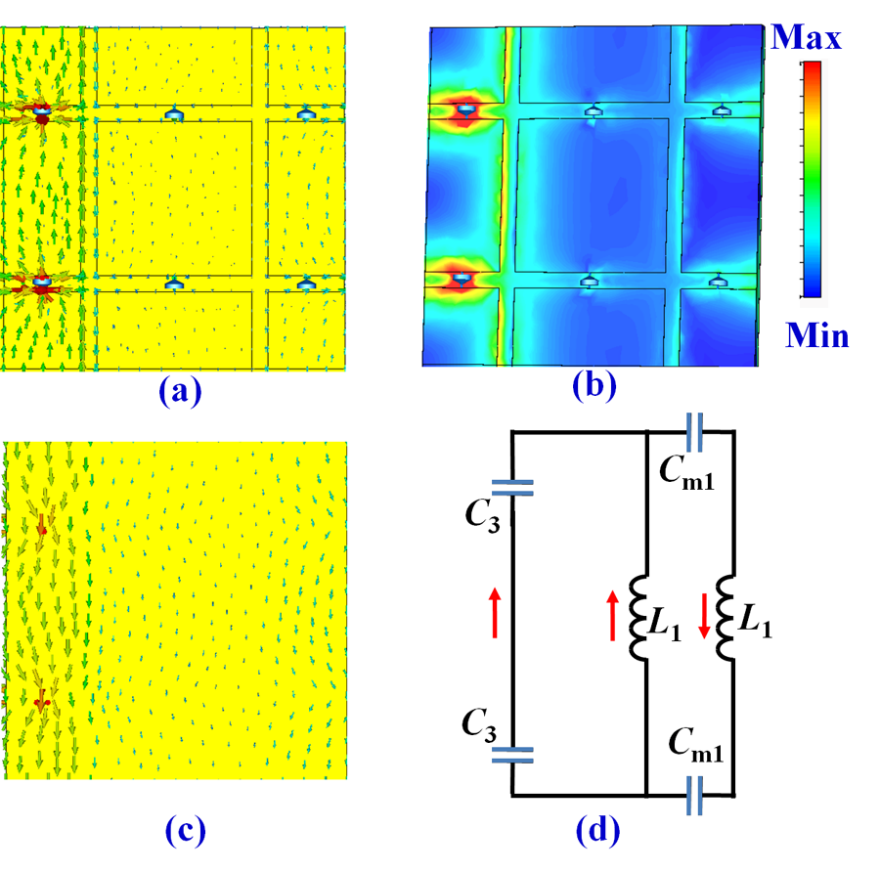


**Figure S4.** Distribution of induced surface currents on (a) front and (c) back metallic layers, and (b) induced magnetic field at the lowest absorption frequency. (d) Reduced equivalent circuit model for the lowest resonance (n = 3).

By applying Eqs. (S4), (S5) and (S7)-(S11), the derived results for equivalent circuit models in Figs. S2(d), S3(d) and S4(d) are shown in Table S2.

**Table S2. Calculated absorption frequencies for triple-band MPA.**

| Number of modes | *w_n_*  (mm) | *l_n_*  (mm) | *C*_n_  (pF) | $\boldsymbol{C}_{\boldsymbol{0}\boldsymbol{n}}^{\boldsymbol{'}}$  (pF) | *C*_mn_  (pF) | *L*_n_  (nH) | *α*_n_ | $\boldsymbol{f}_{\boldsymbol{n}}^{\boldsymbol{'}}$  (MHz) |
| --- | --- | --- | --- | --- | --- | --- | --- | --- |
| n = 1 | 29 | 29 | 47 | 33.5 | 4.57 | 8.61 | 0.4 | 286.0 |
| n = 2 | 14.5 | 29 | 24 | 10.0 | 2.29 | 11.85 | 0.4 | 435.5 |
| n = 3 | 14.5 | 29 | 120 | 60.0 | 2.29 | 11.85 | 0.4 | 186.9 |

**References**

^1^Mohan, S. S. The design, modeling and optimization of on-chip inductor and transformer circuits. *Ph. D. thesis* (Stanford University, 1999).

^2^Zhou, J., Economou, E. N., Koschny, T. & Soukoulis, C. M. Unifying approach to left-handed material design. *Opt. Lett.* **31**, 3620 (2006).

^3^Viet, D. T., Hieu, N. V., Lam, V. D. & Tung N. T. Isotropic metamaterial absorber using cut-wire-pair structures. *Appl. Phys. Express* **8**, 032001 (2015).
